# Effectiveness of an outbreak dose of mumps-containing vaccine in two First Nations communities in Northern Ontario, Canada

**DOI:** 10.1080/21645515.2020.1870909

**Published:** 2021-07-22

**Authors:** Wallis Rudnick, Sarah Wilson, Jo Ann Majerovich, Michelle Haavaldsrud, Marene Gatali, Cai-Lei Matsumoto, Shelley Deeks

**Affiliations:** a Canadian Field Epidemiology Program, Public Health Agency of Canada, Ottawa, Ontario, Canada; b Public Health Ontario, Toronto, Ontario, Canada; c First Nations and Inuit Health – Ontario Region, Indigenous Services Canada, Ottawa, Ontario, Canada; d Sioux Lookout First Nations Health Authority, Sioux Lookout, Ontario, Canada

**Keywords:** Mumps, measles-Mumps-Rubella Vaccine, immunization, outbreak dose, vaccine Effectiveness

## Abstract

Between 18 Dec 2017 and 27 June 2018, a mumps outbreak occurred in two Canadian Indigenous communities. An outbreak dose of mumps-containing vaccine was offered as part of control measures. We conducted a cohort study and survival analysis to describe the outbreak and evaluate the outbreak dose, extracting vaccination information on all community members (n = 3,135) from vaccination records. There were 70 mumps cases; 56% had received two pre-outbreak vaccine doses. Those who received a pre-outbreak dose more distantly had higher rates of mumps compared to those with more recent doses (adjusted hazard ratio = 3.4 (95%CI: 0.7–20.6) for receipt >20 years before vs. receipt ≤3 years). During the outbreak, 33% (1,010/3,080) of eligible individuals received an outbreak dose. The adjusted hazard ratio for no outbreak dose receipt was 2.7 (95%CI: 1.0–10.1). Our results suggest that an outbreak dose of mumps-containing vaccine may be an effective public health intervention, but further study is warranted.

## Introduction

Mumps is an infectious, vaccine-preventable disease that spreads via saliva and respiratory droplets.^1^ Although the classic presentation of mumps infection is acute parotitis, about one in five infections are asymptomatic and 40% to 50% are associated with nonspecific or respiratory symptoms.^2^

In Canada, a mumps vaccine was licensed in 1969 and routine vaccination programs have resulted in a dramatic drop in the number of cases.^1,3,4^ In Ontario, a one-dose measles, mumps, rubella (MMR) vaccine program was implemented in 1975, followed by a two-dose program in 1996 (Figure S1). Estimates of vaccine effectiveness (VE) for mumps-containing vaccine vary considerably: 49% to 91% for one dose and 31% to 97% for two doses.^1,5–7^ VE estimates can be influenced by a number of factors, including intensity of exposure in outbreak settings and waning immunity. Classic and often high profile outbreaks have occurred in institutional settings, such as universities and military facilities.^7–9^ However, community-wide outbreaks also occur and can be difficult to control.^10,11^

Between Dec 18, 2017, and Jun 27, 2018, a mumps outbreak occurred in two remote Indigenous communities in Northern Ontario with a combined population of 3,135. The communities are related by family ties and geographical proximity; roughly 100 kilometers of bush separates the communities. Both are only accessible by plane or ice road in the winter. Primary and urgent health care are provided at a nursing station in each community. At the invitation of the two communities and in consultation with the Northwestern Public Health Unit, the local Indigenous Health Authority, Public Health Ontario and Ontario’s Ministry of Health, an ‘outbreak-dose’ of mumps-containing vaccine was offered to individuals aged 8 to 48 years, including those with two prior doses, as part of the outbreak response.

Our objectives were to: 1) describe the outbreak and the public health response; 2) evaluate the effectiveness of the outbreak dose in this setting; and, 3) evaluate the incremental effectiveness of a third dose of mumps-containing vaccine in the outbreak.

## Materials and methods

### Design

We conducted a population-based cohort study and survival analysis to describe the outbreak of mumps and to evaluate the outbreak dose.

### Cohort

The cohort included residents of Communities A and B. We excluded individuals who were in the communities transiently, such as healthcare workers on two-to-three week rotations. We created the cohort by extracting a population list from the communities’ vaccination record database. The extracted lists were validated for completeness by community leadership. We extracted demographic and vaccination information from the database.

### Pre-outbreak vaccination status

Both communities use a vaccination record database to track vaccinations received by community members. All community members are included in the database. Information about vaccinations received prior to implementation of the database in 1995 was migrated to the vaccination record database upon implementation. Vaccinations received outside of the community are not systematically captured.

For all individuals in the cohort, we extracted dates of receipt for all mumps-containing vaccines received prior to the start of the outbreak, hereafter referred to as “pre-outbreak” doses. If an individual had received a dose of mumps-containing vaccine prior to their first birthday or within 28 days following another dose, these invalid doses were excluded, but the individual was retained in the analysis. We conducted chart reviews on a purposive selection of records to confirm the accuracy of the vaccination database.

### Targeted outbreak response

**Figure 1.**
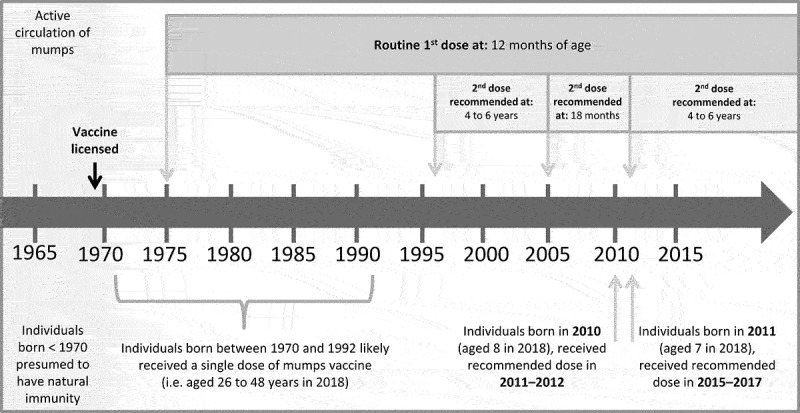
Changes in recommended mumps vaccine schedule in Ontario, Canada.

As part of the outbreak response, an MMR vaccine dose was offered as a “targeted outbreak response” to individuals aged 8 to 48 years from both communities, regardless of their past vaccination history, unless they were a confirmed case of mumps, were confident they had received an MMR vaccine within the last 28 days, or had medical contraindications. The lower end for the targeted age range was selected based on vaccination schedule considerations. Due to changes in Ontario’s vaccination schedule in 2011, children aged 8 and older likely received their second MMR vaccine dose at 18 months while children under 8 years would have received their second dose at ages 4 to 6 years (Figure 1). The upper age limit was selected to reflect the Canadian Immunization Guide’s recommendations to consider people born before 1970 as immune.^12^

The targeted outbreak response supplemented opportunistic vaccination at the time of healthcare encounters for other reasons. In addition, nurses systematically reviewed charts to identify and recall individuals who were not up to date with their vaccinations. Mass vaccination clinics were held in locations such as the community school, band office, and the Northern Store on March 8, 2018 in Community A and March 7–9, 2018 in Community B. Vaccination was only one component of the outbreak response (Table S1).

### Mumps case definition

We used the provincial confirmed mumps case definition and modified the provincial probable case definition to include individuals with clinically compatible signs and symptoms (i.e., parotitis) but with negative laboratory test results (Table S2). This modification was made to improve sensitivity and in recognition of the challenge of mumps diagnostic testing in highly vaccinated populations.^13^ We included as cases all community residents meeting the confirmed and probable case definitions with symptom onset during the outbreak period. The outbreak period differed between the communities and was defined as the date of the first case’s symptom onset to the date of the last case’s symptom onset (Dec 18, 2017–Mar 30, 2018, for Community A; Jan 15, 2018–Jun 27, 2018, for Community B).

### Outbreak dose definition

We defined an ’outbreak dose’ as any dose of mumps-containing vaccine received during the outbreak period. We excluded any doses of mumps-containing vaccine received prior to an individual’s first birthday and doses received within 28 days following another dose.

### Analyses

#### Descriptive epidemiology

We described the outbreak and characteristics of the cases and non-cases prior to the outbreak (for list, see Table 1). We calculated attack rates per 1,000 person-days. Bivariable analyses were performed using Fisher’s exact or Wilcoxon rank sum test, as appropriate. We calculated unadjusted hazard ratios using Firth’s penalized-likelihood bias reduction method for Cox regression, which is a method for dealing with sparse data in survival analyses.

**Table 1.** Population characteristics of Communities A and B at the start of the outbreak, crude attack rates, and unadjusted hazard ratios

Characteristics of the population prior to the outbreak		Cases* /Population	Crude Attack Rate (per 1000 persons)	Unadjusted Hazard Ratio (95% CI)	P-value
Overall		70/3135	22.3		
Age group^†^	<1 year	2/55	36.4	2.6 (0.5–9.3)	.23
	1 to <7 years	4/473	8.5	0.5 (0.2–1.6)	.28
	7 to <18 years	22/788	27.9	1.6 (0.7–3.7)	.26
	18 to <30 years	18/639	28.2	1.6 (0.8–3.9)	.22
	30 to <49 years	16/700	22.9	1.2 (0.5–2.9)	.69
	≥49 years	8/480	16.7	REF	
Gender^‡^	Male	47/1621	29.0	2.0 (1.2–3.5)	.005
	Female	23/1513	15.2	REF	
Community	B	51/2129	24.0	1.0 (0.6–1.8)	.89
	A	19/1006	18.9	REF	
Number of pre-outbreak dose(s) of vaccine received:	≥3 doses	0/54	0	0.8 (0.006–6.5)	.86
	2 doses	39/1445	27.0	2.3 (1.1–5.8)	.03
	1 dose	25/1086	23.0	1.9 (0.8–4.8)	.14
	0 doses	6/550	10.9	REF	
Timing of pre-outbreak dose(s) of vaccine doses^§^	Most recent dose: Received ≥20 years ago	32/962	33.3	2.7 (1.1–8.3)	.03
	Received ≥10 to <20 years ago	21/774	27.1	2.2 (0.9–7.1)	.10
	Received ≥3 to <10 years ago	7/478	14.6	1.3 (0.4–4.6)	.65
	Received <3 years ago	4/371	10.8	REF	

*Confirmed and probable. ^†^Age at the start of outbreak. ^‡^Gender unknown for one individual.

^§^Individuals who received no pre-outbreak doses of mumps-containing vaccine not shown.

#### Outbreak dose evaluation

We included only individuals eligible to receive a valid ‘outbreak dose’ of MMR in the VE analyses. This included all individuals ≥1 year-of-age at the start of the outbreak.

We conducted univariable and multivariable survival analyses using Firth’s method for Cox regression. Confidence intervals were based on the profile penalized log likelihood. Follow-up time started on the first day of the outbreak period and ended on the last day of the outbreak period or on a case’s symptom onset date. We treated receipt of an outbreak dose as a time-varying covariate (Figure S1).

In the survival analysis, to account for immune response, we excluded the 14 days following the receipt of a pre-outbreak dose from the at-risk period for individuals who received a pre-outbreak dose within 14 days of the start of the outbreak (Figure S1). For those who received an outbreak dose, the post-outbreak-dose at-risk period began 14 days following receipt of an outbreak dose (Figure S1). For individuals who went on to develop mumps, we excluded any ‘outbreak dose’ received in the 14-day period prior to, or after, symptom onset. We conducted sensitivity analysis with shorter and longer periods (7 days and 28 days).

In the multivariable survival analysis, our primary variable of interest was receipt of an outbreak dose. Our initial model was selected using subject-matter knowledge. This model included receipt of an outbreak dose, age, gender, and time since most recent receipt of pre-outbreak vaccine. The events-per-degree-of-freedom (EPF) in this model was 6.9. EPF is a ratio that compares the number of events in a model to the number of predicted parameters; higher EPFs will produce more reliable estimates of parameters. All covariables were retained in the final model since each variable maintained the precision of the primary estimate.

We calculated VE for receipt of an outbreak dose as 1-hazard ratio. We conducted sensitivity analysis by excluding probable cases and by examining effects by age group and by pre-outbreak vaccination status (see Table S3 for list of additional models and associated estimates of VE).

We completed all analyses using Stata 13.1 (stata.com), SAS 9.3 (sas.com) or R 3.3.2 (r-project.org). *P*-values < 0.05 were considered significant.

### Ethics

The outbreak response and evaluation were undertaken with the approval and support of community leadership, including the Chiefs, Band Council Members and the Health Directors. The communities provided their data as part of the response efforts so at-risk individuals could be identified and offered vaccination and so the response could be evaluated. Community leadership supported the sharing of their experiences and the publication of their data. This evaluation was conducted as part of the outbreak response, was considered non-research and was not subject to an institutional review.

## Results

### Description of the outbreak

**Figure 2.**
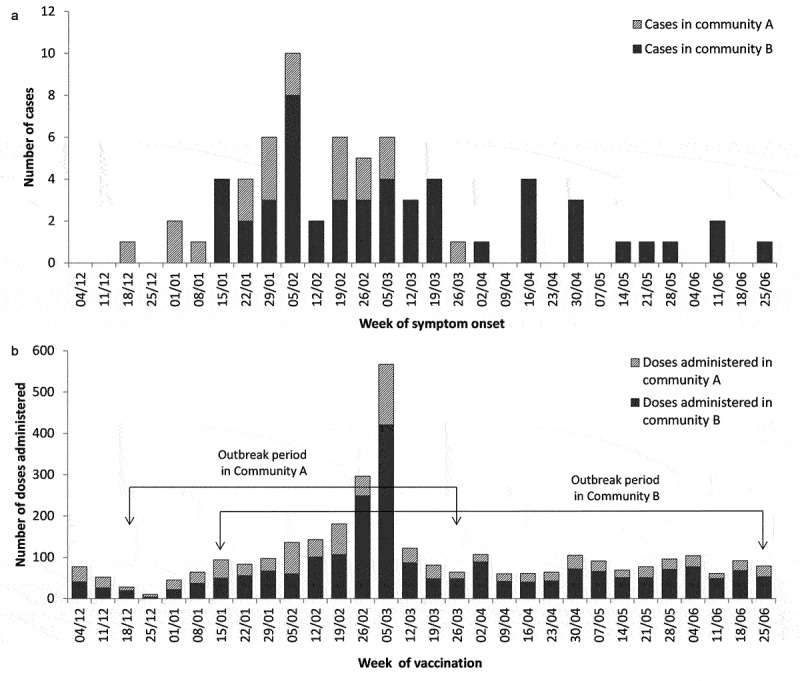
[Panel A] Epi-curve for the 70 confirmed and probable cases of mumps occurring in Communities A and B: Dec 18, 2017, to June 27, 2018. [Panel B]: MMR vaccine doses administered in Communities A and B: Dec 18, 2017, to Jun 27, 2018.

Between Dec 18, 2017, and Jun 27, 2018, there were 70 mumps cases (52 confirmed and 18 probable) identified as part of the outbreak (crude attack rate: 22.3/1,000 persons) (Figure 2). The outbreak started in Community A and then approximately 1 month later began in Community B, although no direct epidemiologic link could be made between cases in the two communities. There was one hospitalization and no deaths. Five cases (5/70, 7%) had complications including orchitis (n = 3), oophritis (n = 1), and neurological symptoms (slurred speech, tunnel vision, hearing loss; n = 1). Routine mumps genotyping confirmed that cases were infected with a strain of mumps genotype G that has been circulating in Canada for many years, including the period of increased mumps activity in 2017.

### Population and case characteristics

The median age of cases was 24 years (age range at symptom onset: 10 months to 62 years), slightly older than the median age of non-cases (22 years, range: 0 to 91 years). Males accounted for 67% of cases compared to 51% of non-cases (*P* < .009).

Among the 70 mumps cases, 91% had received ≥1 pre-outbreak MMR vaccine dose: 36% (25/70) had received one dose, and 56% (39/70) had received two. Among non-cases, 82% had received ≥1 pre-outbreak MMR vaccine dose: 35% (1,061/3,065) had received one dose, 46% (1,406/3,065) two doses and 2% (54/3,065) more than 2 doses.

Among cases who received ≥1 pre-outbreak dose, the median time since receipt of a dose was 20 years (interquartile range (IQR): 12–26 years) compared to 15 years (IQR: 7–22 years) among non-cases.

### Pre-outbreak characteristics

In the univariable analysis of risk factors (Table 1), crude attack rates were significantly higher among males, those with remote receipt of pre-outbreak vaccine (>20 years) and those with two doses of vaccine. Stratification of the data by age group, number of pre-outbreak doses and time since most recent pre-outbreak dose showed strong associations between these variables (data not shown).

The attack rate for individuals with no pre-outbreak doses of mumps-containing vaccine was lower than that observed among those with one or two doses of vaccine. Among the six cases with no pre-outbreak doses, two occurred among infants and three occurred among individuals born before 1970. The final case was in a child in the 7 to <18 year age range. Half (51%) of those with no pre-outbreak doses were born before 1970.

### Outbreak dose evaluation

Of the 3,135 individuals in the communities, 3,080 (98%) were eligible to receive a valid dose of MMR vaccine (≥1 year of age at the start of the outbreak) and were included in the evaluation of the outbreak dose. Of these, 1,010 (33%) received an outbreak dose (Figure 2; 30 received two doses). Fifteen percent of eligible individuals received an outbreak dose during the mass clinics (representing 45% of all outbreak doses administered). Individuals with no history of vaccination were more likely than others to receive an outbreak dose (38% (186/495) vs 32% (824/2585), *P* = .01). Among those specifically targeted for an outbreak dose (those aged 8 to 48 at the start of the outbreak), 38% (780/2,050) received at least one dose. This varied by pre-outbreak vaccination status. There was 54% uptake among those with no prior doses, 48% among those with one pre-outbreak dose, 31% among those with two pre-outbreak doses, and 25% among those with more than two pre-outbreak doses.

There were 68 mumps cases among individuals age-eligible for MMR vaccine (crude attack rate = 22.1/1,000 persons). Three cases occurred in individuals who had received an outbreak dose; one case (born before 1970) had received no pre-outbreak doses, one case (30 to <49 years) had one pre-outbreak dose and one case (18 to <30 years) had two pre-outbreak doses of vaccine. We completed a chart review and none had any underlying medical condition that would influence the immune response to vaccination or evidence of vaccine strain following genotyping.

In the final adjusted model, among the eligible population, the hazard ratio comparing no receipt of an outbreak dose to receipt of an outbreak dose was 2.7 (95%: 1.0–10.1) (Table 2). The risk of developing mumps was significantly higher among males. The associated estimated VE for receipt of an outbreak dose was 63% (95%CI: 0–90%).

**Table 2.** Unadjusted and adjusted hazard ratios for becoming a case of mumps among residents eligible to receive mumps vaccine (aged ≥1 year) during the outbreak, 18-Dec-2017 to 27-June-2018

		Penalized-likelihood Cox models		
Variable		HR ^‡^ estimate	95% Confidence Limits	*P*-value
Unadjusted estimates				
Outbreak dose*	No outbreak dose	2.5	(0.9–9.4)	.08
	Receipt of outbreak dose	REF		
Age group	1 to <7 years	0.5	(0.2–1.6)	.28
	7 to <18 years	1.6	(0.7–3.6)	.26
	18 to <30 years	1.6	(0.8–3.9)	.22
	30 to <49 years	1.2	(0.5–2.9)	.69
	≥49 years	REF		
Gender	Male	2.1	(1.3–3.6)	.004
	Female	REF		
Community	B	1.0	(0.6–1.8)	.99
	A	REF		
Number of previous pre-outbreak dose(s) of vaccine	≥3 doses	1.0	(0.008–9.4)	1.0
	2 doses	3.0	(1.3–9.2)	.01
	1 dose	2.4	(1.0–7.7)	.06
	0 doses	REF		
Timing of pre-outbreak dose(s) of vaccine^†^	Most recent dose:			
	Received ≥20 years ago	2.7	(1.1–8.3)	.03
	Received ≥10 to <20 years ago	2.2	(0.9–7.1)	.10
	Received ≥3 to <10 years ago	1.3	(0.4–4.6)	.65
	Received <3 years ago	REF		
Adjusted estimates (multivariable model^§^)				
Outbreak dose *	No outbreak dose	2.7	(1.0–10.1)	.06
	Receipt of outbreak dose	REF		
Age group	1 to <7 years	1.0	(0.2–5.4)	.97
	7 to <18 years	3.0	(1.0–9.3)	.05
	18 to <30 years	1.4	(0.6–3.5)	.44
	30 to <49 years	0.8	(0.3–2.0)	.57
	≥49 years	REF		
Gender	Male	2.0	(1.2–3.5)	.006
	Female	REF		
Timing of previous dose(s) of vaccine	No pre-outbreak vaccine	0.9	(0.2–4.8)	.91
	Most recent dose:			
	Received ≥20 years ago	3.4	(0.7–20.6)	.13
	Received ≥10 to <20 years ago	1.4	(0.3–7.9)	.70
	Received ≥3 to <10 years ago	0.6	(0.2–4.8)	.61
	Received <3 years ago	REF		

*Time-varying variable: the post-vaccine at-risk period begins 14 days following receipt of the outbreak dose.

^†^Individuals who received no pre-outbreak doses of mumps-containing vaccine not shown.

^‡^HR = Hazard ratio.

^§^Multivariable model includes receipt of outbreak dose, age group, gender, and timing of previous dose(s) of vaccine.

Among the 1,445 individuals who had received two pre-outbreak doses of mumps-containing vaccine, the VE estimate for a third dose was 69%, but confidence intervals were wide (adjusted HR = 3.3, 95%CI: 0.8–29.9).

In the sensitivity analysis, when the post-vaccine at-risk period was decreased to 7 days, the VE estimate decreased (30%, unadjusted HR = 1.4 (95%CI: 0.5–5.4)). When the post-vaccine period was increased to 28 days, estimates of VE increased but confidence intervals were very wide (85%, unadjusted HR = 6.6 (95%CI: 0.8–851.4)) (Table S3). When the analysis only included confirmed cases, the VE estimate decreased slightly (56%, unadjusted HR = 2.3, 95%CI: 0.5–21.2).

## Discussion

Despite routine vaccination programs, periodic outbreaks of mumps occur in both closed^14,15^ and community-based settings^10,11,16,17^ which present different challenges for outbreak control. This outbreak involved two remote close-knit communities, with very high attack rates across a range of ages from under 1 to 62 years and with less than 50% two-dose mumps coverage prior to the outbreak. During the outbreak, 33% of the eligible population received a dose of vaccine with 15% receiving an outbreak dose during the 3 days of the mass vaccination clinics. Although this was a community-based outbreak, it has some features that are similar to outbreaks in closed settings, such as a clearly identified population, centralized nursing staff, and a single health information system.

The results of our unadjusted analysis suggest that being male, receiving a mumps-containing vaccine more distantly, and having two previous doses of vaccine were significantly associated with becoming a mumps case. Not receiving an outbreak dose was associated with a 2.5-fold higher risk of mumps, but this finding was not statistically significant. However, in our adjusted analysis, the only variable that remained significantly associated with becoming a mumps case was male sex. Although there appeared to be a dose response with time since last dose of vaccine in the adjusted analysis, this finding did not reach statistical significance. Similarly, in the adjusted analysis, individuals who did not receive an outbreak dose were 2.7 times more likely to become a case compared with those who did receive a dose, but this finding did not reach statistical significance. As such, our findings suggest that in addition to sex, waning immunity may play a role in mumps transmission dynamics, and that the use of mumps vaccine during an outbreak may impact these dynamics, although further studies are needed to confirm this.

Although our findings on the impact of an outbreak dose did not reach statistical significance, they align with an accumulating body of evidence on the importance of waning vaccine-induced immunity in outbreaks. Waning of vaccine-derived immunity has been suggested as the primary driver of mumps outbreaks in settings with very high two-dose coverage,^18–20^ yet it is notable that, even in this outbreak with less than 50% two-dose mumps coverage prior to the outbreak, increased time since last mumps-containing vaccine dose was associated with increased risk of mumps, regardless of pre-outbreak vaccination status.

Among those for whom the outbreak dose was a third dose of mumps-containing vaccine, the overall VE of the third dose was 69% with a 14-day post-vaccine window and 85% with a 28-day post-vaccine window. Although point estimates must be interpreted cautiously due to the wide confidence intervals, the estimates of the incremental effectiveness of a third dose are generally consistent with the estimates reported by Cardemil et al. from a 2017 university outbreak.^18^ They found that the incremental vaccine effectiveness ranged from 60% to 78%, depending on the length of the post-vaccine window (range from 7 to 28 days).^18^ Our estimates with a 7-day window (30%) were much lower, possibly due to the differences in intensity of exposure between the two outbreaks prior to the launch of the respective outbreak dose campaigns.

In Canada, it is assumed that individuals born before 1970 have naturally-acquired immunity to mumps due to the wide circulation of the virus prior to vaccine introduction;^1^ however, in this outbreak 11% (8/70) of cases occurred in individuals born before 1970. This may be related to the force of infection of the outbreak or it may reflect a different age profile of mumps susceptibility in this community. This later hypothesis raises the question as to whether the 1970 cutoff is appropriate in isolated settings where active mumps circulation may have ended earlier than in other regions of Canada. Despite cases in this age group, those born before 1970 had one of the lowest attack rates among any of the age groups and likely explain why the attack rate among those with no pre-outbreak doses was lower than expected. Another explanation for the low attack rate among those with no pre-outbreak doses includes potential misclassification due to missing vaccine doses in the database. This would be more likely among individuals who had spent time away from the communities and those who received vaccine prior to the implementation of the database in 1995. Vaccinations administered off-reserve are not captured systematically.

In this outbreak, the decision to implement a targeted outbreak dose program was based on the early epidemiology, considerations of the change in Ontario MMR vaccine policy in 2011 and the accumulating evidence for waning of vaccine-derived immunity. At the time of this outbreak, a position statement on the use of an outbreak dose (including a third dose) of mumps-containing vaccine had not yet been issued by Canada’s National Advisory Committee on Immunization (NACI) although an existing NACI recommendation supported the use of a second dose of mumps-containing vaccine for under-immunized groups, including adults, during outbreaks.^9^ The third dose recommendation of the Advisory Committee on Immunization Practices (ACIP) had been recently released but the subsequent implementation guidance of the Centers for Disease Control (CDC)^7^ was not available. This revised CDC guidance would have supported offering a third dose to age-eligible community members based on a decision matrix encompassing risk and evidence of transmission and the additional consideration described as “the setting is known to be high risk for transmission based on previously reported outbreaks, e.g., fraternities, sport teams, or close knit communities.”^20^

The strengths of this outbreak evaluation included: 1) the use of an outbreak dose in a real-world setting with high attack rates across a range of ages, and 2) the existence of a vaccination database for all members of the community which was instrumental to response efforts and to conducting the subsequent evaluation. However, a number of limitations should be noted. This evaluation illustrates the challenges in disentangling important predictors of mumps infection in community-based evaluations. Our variables of interest in this analysis, notably age, time since last dose, and pre-outbreak vaccination status, were not independent, making it challenging to draw definitive conclusions about these variables in isolation. Despite a high attack rate, our small sample size means that confidence intervals remain wide and we are limited in our ability to separate the effects of covariables. This outbreak occurred in a real-world setting and our study design was observational in nature and thus, subject to potential bias. Vaccinated individuals may be systematically different from those who are not vaccinated (healthy user effect^21^). There may be unmeasured confounders associated with both receipt of vaccination and risk of mumps in this outbreak including receipt of other interventions included in the response efforts. If individuals who were in contact with mumps and therefore at higher risk of infection were more likely to seek out vaccination, VE would be underestimated. Some vaccinations may not have been captured in the database. As the vaccination campaign was only one component of response efforts, we are not able to draw conclusions about the effects of the vaccination campaign on the overall progress of the outbreak.

## Conclusions

Our evaluation suggests that an outbreak dose of mumps-containing vaccine may be an effective public health intervention during an outbreak, but this requires confirmation in future studies as our results did not reach statistical significance. This evaluation illustrates the potential for very high attack rates across a range of ages when mumps is introduced within close-knit communities and the challenges in disentangling important predictors of mumps infection in community-based evaluations. It also suggests that despite centralized delivery of publicly-funded vaccine, a clear target population, and the availability of a centralized vaccination database, mounting an outbreak vaccination campaign and attaining high uptake of an outbreak dose can be challenging. Additional studies are required to both confirm our findings and identify factors associated with increasing vaccine uptake during community-based mumps outbreaks.

## Supplementary Material

Supplemental Material
