# Supplementary figures and images for: Effectiveness of an outbreak dose of mumps-containing vaccine in two First Nations communities in Northern Ontario, Canada
Source: Hum Vaccin Immunother. 2021 Jul 22;18(1):1870909. doi: 10.1080/21645515.2020.1870909 (PMC8920173; doi:10.1080/21645515.2020.1870909)

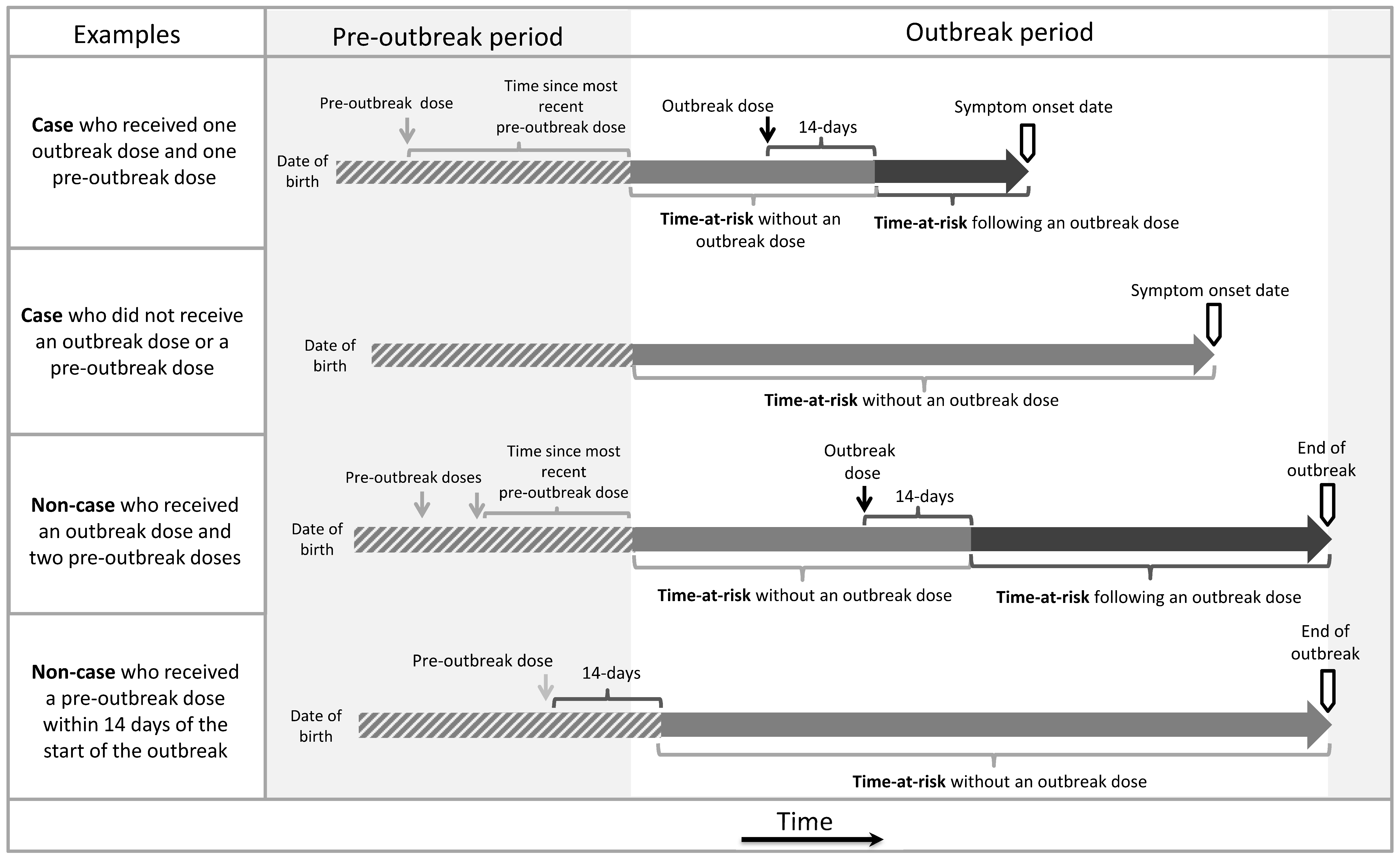

Supplement: Supplemental Material [file KHVI_A_1870909_SM4719.tiff]
